# Epigenetic Silencing of Immune-Checkpoint Receptors in Bone Marrow- Infiltrating T Cells in Acute Myeloid Leukemia

**DOI:** 10.3389/fonc.2021.663406

**Published:** 2021-05-04

**Authors:** Ramin Radpour, Miriam Stucki, Carsten Riether, Adrian F. Ochsenbein

**Affiliations:** ^1^ Tumor Immunology, Department for BioMedical Research (DBMR), University of Bern, Bern, Switzerland; ^2^ Department of Medical Oncology, Inselspital, Bern University Hospital, University of Bern, Bern, Switzerland

**Keywords:** acute myeloid leukemia, immune-checkpoints, immunotherapy, leukemia stem cell (LSC), CD8^+^ T cell, CD4^+^ T cell, epigenetics (chromatin remodelling), histone (de)acetylation

## Abstract

**Background:**

Immune-checkpoint (IC) inhibitors have revolutionized the treatment of multiple solid tumors and defined lymphomas, but they are largely ineffective in acute myeloid leukemia (AML). The reason why especially PD1/PD-L1 blocking agents are not efficacious is not well-understood but it may be due to the contribution of different IC ligand/receptor interactions that determine the function of T cells in AML.

**Methods:**

To analyze the interactions of IC ligands and receptors in AML, we performed a comprehensive transcriptomic analysis of FACS-purified leukemia stem/progenitor cells and paired bone marrow (BM)-infiltrating CD4^+^ and CD8^+^ T cells from 30 patients with AML. The gene expression profiles of activating and inhibiting IC ligands and receptors were correlated with the clinical data. Epigenetic mechanisms were studied by inhibiting the histone deacetylase with valproic acid or by gene silencing of *PAC1*.

**Results:**

We observed that IC ligands and receptors were mainly upregulated in leukemia stem cells. The gene expression of activating IC ligands and receptors correlated with improved prognosis and vice versa. In contrast, the majority of IC receptor genes were downregulated in BM-infiltrating CD8^+^ T cells and partially in CD4^+^ T cells, due to pathological chromatin remodeling *via* histone deacetylation. Therefore, treatment with histone deacetylase inhibitor (HDACi) or silencing of *PAC1*, as a T cell-specific epigenetic modulator, significantly increased the expression of IC receptors and defined effector molecules in CD8^+^ T cells.

**Conclusions:**

Our results suggest that CD8^+^ T cells in AML are dysfunctional mainly due to pathological epigenetic silencing of activating IC receptors rather than due to signaling by immune inhibitory IC receptors, which may explain the limited efficacy of antibodies that block immune-inhibitory ICs in AML.

## Introduction

Acute myeloid leukemia (AML) is a hematologic malignancy with a poor clinical prognosis. It arises from clonal expansion of oncogene-transformed hematopoietic stem and progenitor cells, known as leukemia stem cells (LSCs) (1). LSCs are therapy resistant and evade direct killing by immune cells due to diverse escape mechanisms. Therefore, LSCs are the leading cause of relapse after initial successful chemotherapy (2).

Immune cells are part of the bone marrow (BM) microenvironment and interact with the leukemia stem/progenitor cells. Clinical observations and experimental evidence suggest that myeloid leukemia is regulated by the immune system (3). Tumor cells, including LSCs, can suppress effective tumor-specific T cell responses. LSCs escape the immune control by natural killer (NK) cells and CD8^+^ T cells through the expression of immune-inhibitory molecules by downregulating of critical molecules/pathways involved in immune recognition, or by the expression of “don’t eat me” signals, such as CD47 (4). Immune-checkpoints (ICs) are essential regulators of the immune system, through activation (stimulatory checkpoint molecules) or inhibition (inhibitory checkpoint molecules) of immune cells (5, 6). The balance of stimulatory and inhibitory ligand/receptor interaction determines the amplitude and quality of the antigen-specific immune responses (5). The generation of immune-checkpoint inhibitors (ICIs) that blocks the inhibitory IC receptors, such as cytotoxic T-lymphocyte-associated protein 4 (CTLA-4) or programmed cell death protein 1 (PD-1), has revolutionized the treatment of different solid tumors and lymphomas (7). In contrast, blocking ICs in the treatment of leukemia seems less effective (8–10). This is potentially due to BM-infiltrating T cells expressing other inhibitory receptors, such as T cell immunoglobulin and mucin-domain containing-3 (TIM-3) and Lymphocyte-activation gene 3 (LAG-3) (11). Indeed, understanding the expression signature of ICs on leukemia stem/progenitors and paired lymphocytes in the BM is crucial for the development of novel immunotherapy approaches.

In the present study, we performed a comprehensive expression analysis of important IC ligand/receptor pairs in AML stem and progenitor cells in parallel with paired BM-infiltrating lymphocytes. We observed that gene expression of IC ligands and receptors were mainly upregulated in leukemia stem and progenitor cells (LSPCs). A high gene expression of IC ligands and receptors with an activation function or low gene expression of IC ligands and receptors with inhibition function in LSCs correlated with better overall survival. In contrast, the majority of IC receptor genes were downregulated in BM-infiltrating CD8^+^ T cell and partially in CD4^+^ T cells. We identified pathological histone deacetylation as the main cause for the downregulation of IC receptors in CD8^+^ and CD4^+^ T cells. Therefore, treatment with histone deacetylase inhibitor (HDACi) significantly increased the expression of IC receptors on T cells in AML at both gene and protein levels. Furthermore, the expression of the phosphatase *PAC1*, a key T cell-specific epigenetic modulator, negatively correlated with the expression of IC receptors in T cells from AML patients. Consequently, silencing of the *PAC1* gene significantly increased the expression of different ICs on T cells. These findings could have potential implications for the design of immunotherapies that target AML and/or AML LSCs.

## Materials and Methods

### Patients

Blood and BM aspirates from patients diagnosed with AML were prospectively collected at the Department of Medical Oncology, University Hospital Bern. Thirty patients were selected from this repository based on the FACS immune-phenotype of the AML cells and the risk category. Risk categories of AML patients were determined according to the defined molecular profile of patients (guidelines for Dutch-Belgian Hemato-Oncology Cooperative Group (HOVON) and Swiss Group for Clinical Cancer Research (SAKK)). BM aspirates performed as a staging procedure in patients with lymphoma that did not have a pathological infiltration in the aspirate nor the biopsy were used as controls.

### FACS-Purification of Stem/Progenitors and Paired CD4^+^/CD8^+^ T Cells

LSCs were defined as CD45^+^Lin^-^CD90^-^CD38^-^CD34^+^, leukemic progenitor cells (LPCs) as CD45^+^Lin^-^CD90^-^CD38^+^CD34^+^, hematopoietic stem cells (HSCs) as CD45^+^Lin^-^CD90^+^CD38^-^CD34^+^ and normal progenitors (HPCs) as CD45^+^Lin^-^CD90^+^CD38^+^CD34^+^. CD4^+^ T lymphocytes were CD45^+^Lin^+^CD4^+^. CD8^+^ T lymphocytes were CD45^+^Lin^+^CD8^+^. Lineage positive cells were defined by the expression of CD2, CD3e, CD14, CD16, CD19, CD56 or CD235. All cell populations were FACS-purified according to their immunophenotype using a FACS ARIA III (BD Biosciences, USA).

### Antibodies and Flow-Cytometry

αCD2-biotin (clone: RPA-2.10), αCD14-biotin (clone: HCD14), αCD16-biotin (clone: 3G8), αCD19-biotin (clone: 561), αCD235a-biotin (clone: HIR2), αCD3-biotin (clone: OKT3a), αCD34-APC (clone: 561), αCD38-PE/Cy7 (clone: HIT2), αCD4-APC/Cy7 (clone: RPA-T4), αCD8a-Pacific Blue (clone: RPA-T8), αCD90-PerCP/Cy5.5 (clone: 5E10), αCD272-FITC (αBTLA; clone: MIH26), αIgG2-FITC (clone: MOPC-173), αCD200R-PE (clone: OX-108), αIgG1-PE (clone: MOPC-21), αCD160-PerCP/Cy5.5 (clone: BY55), αIgM-PerCP/Cy5.5 (clone: MM-30), αCD96-APC (clone: NK92.39), αIgG1-APC (MOPC-21), αCD28-PE/Cy7 (clone: CD28.2), αIgG1-PE/Cy7 (MOPC-21) and αStreptavidin-FITC were from BioLegend, USA. αCD45-PE (clone: HI30) was from eBioscience, USA. αIgG1-PE (clone: IS11-12E4.23.20) was from Miltenyi Biotec, Germany. Samples were analyzed on BD LSRFortessa™ (BD Biosciences, USA). Data was analyzed using FlowJo software v.10.7 (FlowJo, LLC, USA).

### Molecular Profiling

To analyze the interactions of IC ligands and receptors in AML, we performed a detailed transcriptomic analysis in FACS-purified leukemia stem/progenitor cells and paired CD4^+^ and CD8^+^ T cells from 30 patients with AML and seven controls. In total, 148 samples from 30 AML patients and seven controls were analyzed using the human expression array “GeneChip^®^ Human Transcriptome Array 2.0 (HTA 2.0)” (Affymetrix Inc., USA). The HTA 2.0 high-resolution array contains >6.0 million probes covering both coding and non-coding transcripts. This provides an in-depth insight into all coding and non-coding transcripts by providing the coverage and accuracy required to detect all known transcript isoforms produced by a gene. Of note, 146 samples were selected for further analysis because of insufficient mRNA amplification of two LSCs samples; due to limiting cell numbers (patients #7 and #23).

Total RNA was extracted from the FACS-purified samples using RNeasy Micro Kit (QIAGEN, Switzerland). The quantity of extracted RNA was assessed by NanoDrop ND-1000 spectrophotometer (NanoDrop Technologies, Inc., USA) and by Bioanalyzer instrument using the RNA 6000 Pico Chip (Agilent Technologies, Germany). The purified RNA was quantified using the QuantiFluor RNA System (Promega, USA). cDNA was synthesized using the GeneChip^®^ Human Transcriptome Pico Assay 2.0 (Affymetrix Inc., USA). The arrays were hybridized with the biotin-labeled fragments using GeneChip^®^ Hybridization, Wash, and Stain Kit (Affymetrix Inc., USA), and rotated in the hybridization oven for 48 hours at 45°C and 60rpm. The arrays were washed and stained with a streptavidin phycoerythrin conjugate on a GeneChip Fluidics 450 Workstations and scanned on a GeneChip Scanner 3000 7G (Affymetrix Inc., USA). The expression data were acquired using the Affymetrix GeneChip Operating Software (GCOS). HTA annotations supplied by Affymetrix were used as probe identifiers.

### Transcriptomic Data Analysis

HTA data analysis was performed after Robust Multi-Array Analysis (RMA), normalization, and log transformation. Differentially expressed genes were defined according to the following criteria: mean intensity greater than three, fold change greater than 1.5 and *P*-values set to *P*<0.05. Principal component analysis (PCA) was used to map the variations among profiled samples. Unsupervised hierarchical clustering of significant upregulated or downregulated genes was applied using the standard Euclidean’s method and the heat maps were generated according to the standard normal distribution of the values.

### Gene Expression Analyses Using Quantitative RT-PCR (qPCR)

Several target genes were analyzed by real-time (qRT)-PCR. cDNA was synthesized using a High-Capacity cDNA Reverse Transcription Kit (Applied Biosystems, USA). Primers were designed for each gene by Primer3Plus (http://www.bioinformatics.nl/) or using Primerquest Software (Integrated DNA Technologies). The complete sequences of used primers are listed in **Supplementary Table 1**. qRT-PCR was performed using FastStart Universal SYBR^®^ Green 2X PCR Master Mix (Roche, Switzerland). Raw values were normalized using the geometric mean of reference genes (*ACTB* and *GAPDH*). qRT-PCR reactions were performed in replicate using QuantStudio 3 System or ABI Prism 7500 Sequence Detection System (Applied Biosystems).

### Gene Set Enrichment Analysis

Gene set enrichment analysis (GSEA) was performed using GSEA software (Broadinstitute, Cambridge, USA). Enrichment analysis was assessed for 30 IC ligand genes and 30 IC receptor genes, respectively.

### Histone Deacetylase Inhibition

FACS-purified CD4^+^ or CD8^+^ T cells were seeded in 48-well culture plates at a density of 1×10^5^ cells/well in IMDM media (Sigma, Switzerland) supplemented with 10% FCS, 1% Pen-Strep (Sigma, Switzerland), and hIL-2 5u/ml (Prospect). Cells were treated with 1 mM sodium valproate (VPA) (Sigma, Switzerland) or PBS. Then, 24h post treatment, total RNA was extracted and, after cDNA synthesis, qRT-PCR was assessed for a panel of selected genes, as described above. The CD4^+^ or CD8^+^ T cells were harvested 48h post treatment and FACS-analyzed for the protein expression of selected ICs.

### Gene Silencing of *PAC1 (DUSP2)*

The human *PAC1* gene was silenced in isolated human CD8^+^ or CD4^+^ T cells from BM of AML patients using siRNA according to the manufacturer’s instructions (Santa Cruz Biotechnology; cat. sc-39004). Briefly, *siPAC1* or *siCtrl* (scrambled control) was mixed with Lipofectamine LTX (Thermo Fisher Scientific) in serum-free media. Isolated CD8^+^ or CD4^+^ T cells were subsequently treated with transfection complexes in the presence of antibiotic-free growth medium supplemented with 10% FCS and hIL-2 5μ/ml (Prospect) for 48h.

### Statistical Analysis

Statistical analysis for qRT-PCR data and functional studies were performed using GraphPad Prism (GraphPad Software, USA). The Shapiro-Wilk test was used to assess the assumption of normality in the datasets. Gene expression data were analyzed using Student’s t-test (2-tailed). Survival time differences were plotted using Kaplan-Meier curves and analyzed using the log-rank test. The assigned cut-point for the survival analysis was assessed using the X-Tile program based on the mean expression of the immune-checkpoints gene panel. All *P*-values were considered significant when *P*<0.05.

### Data Availability and Resources

All transcriptomic data compiled in this study have been deposited in NCBI GEO under the accession codes: GSE117090.

## Results

### IC Receptors and Ligands Are Upregulated in AML Stem and Progenitors

**Figure 1:**
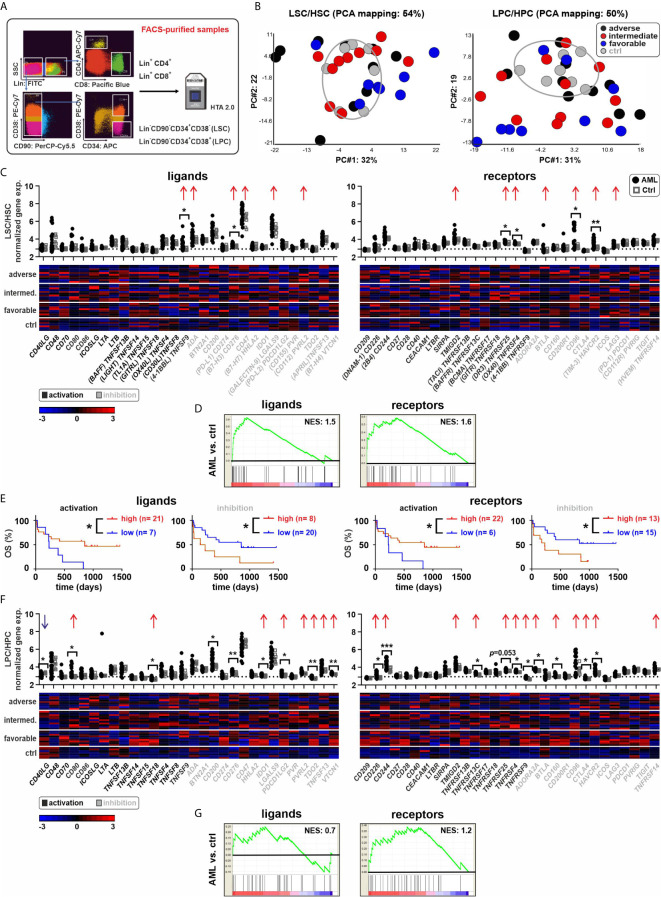
Expression of ICs in AML stem and progenitor cells. **(A)** Experimental setup. Leukemia stem or progenitor cells (LSCs/LPCs), control hematopoietic stem or progenitor cells (HSCs/HPCs), and paired T cells were FACS-purified. **(B)** PCA of LSCs/HSCs and LPCs/HPCs from AML patients and controls. **(C)** Gene expression patterns of ICs in AML LSCs compared to controls (30 ligands and 30 receptors). Dot plots show the gene expression profiles (red arrows showing upregulated ICs and blue arrows indicating downregulated ICs; Fold difference ≥ 1.3). Heatmaps illustrate the expression patterns in respective AML risk groups. **(D)** Gene set enrichment analysis (GSEA) representing the normalized enrichment score (NES) of IC gene sets on LSCs (AML vs. ctrl). **(E)** Kaplan–Meier plots of overall survival (OS) for AML patients in the study cohort according to the gene expression signature of activating or inhibitory ICs in LSCs. **(F)** Gene expression patterns of ICs in AML LPCs compared to controls. Dot plots show the gene expression profiles and heatmaps illustrate the expression patterns in respective AML risk groups. **(G)** GSEA representing the NES of IC gene sets on LPCs (AML vs. ctrl). Statistics: student’s t-test and log-rank test. *P < 0.05, **P < 0.01, ***P < 0.001.

The expression of 30 IC ligand/receptor pairs was analyzed in FACS-purified stem and progenitor cells together with paired lymphocytes (CD4^+^ and CD8^+^ T cells) from BM aspirates of 30 patients with newly diagnosed AML. Seven BM aspirates from lymphoma patients without documented infiltration were used as controls (**Figure 1A**). In total, 146 samples from different cell populations were analyzed. The clinical and molecular characteristics of AML patients are summarized in **Table 1** and the complete characteristics of AML patients and controls have previously been published by our group (12).

**Table 1:** Clinical and molecular characteristics of AML patients in the present study.

Patient ID	Sex	Age (year)	AML risk category	Molecular diagnostic	Cytogenetics	BMblasts (%)
**AML#1**	M	46	Adverse	*FLT3-ITD*	Normal	95
**AML#2**	M	66	Adverse	*FLT3-ITD*	Normal	90
**AML#3**	F	49	Adverse	*FLT3-ITD*	Normal	97
**AML#4**	M	76	Intermediate	*FLT3-ITD, NPM1*	Normal	70
**AML#5**	F	65	Intermediate	*FLT3-ITD, NPM1*	Normal	90
**AML#6**	F	71	Intermediate	*FLT3-ITD, NPM1*	Normal	60
**AML#7**	F	63	Favorable	*NPM1*	Normal	90
**AML#8**	M	54	Favorable	*NPM1*	Normal	20
**AML#9**	F	80	Favorable	*NPM1*	Normal	90
**AML#10**	F	66	Favorable	*AML1-ETO*	t(8;21)	90
**AML#11**	F	40	Favorable	*AML1-ETO*	t(8;21)	70
**AML#12**	M	69	Adverse	*MLL-Rearrangement*	Trisomy 11q23.3	80
**AML#13**	M	39	Intermediate	*MLL-Rearrangement*	t(11;17)(q23; q12-21)	95
**AML#14**	M	32	Favorable	*CBFB/MYH11*	inv(16)	90
**AML#15**	M	73	Favorable	*CBFB/MYH11*	inv(16)	90
**AML#16**	F	30	Favorable	*CEPBA*	Normal	40
**AML#17**	F	46	Intermediate	*JAK2*	Trisomy 8	90
**AML#18**	F	57	Adverse	Normal	CK	40
**AML#19**	M	79	Adverse	Normal	t(4;8)(q21;q22), del(9)(p21), del(18)(q21)	95
**AML#20**	M	60	Intermediate	Normal	Trisomy 13 Trisomy 21	80
**AML#21**	M	70	Intermediate	Normal	Normal	80
**AML#22**	M	69	Adverse	Others	Monosomy 7 (total), 5q31.2 and 5q33	85
**AML#23**	M	59	Intermediate	Normal	Normal	70
**AML#24**	F	40	Adverse	Others	CK	90
**AML#25**	M	69	Adverse	Normal	CK with Monosomy 5q,7, 14, 15, 16, 18	80
**AML#26**	M	40	Favorable	Normal	t(15,17)	90
**AML#27**	F	75	Intermediate	Normal	Normal	20
**AML#28**	M	59	Adverse	Normal	Monosomy 7q31.2	80
**AML#29**	F	20	Intermediate	Normal	Normal	60
**AML#30**	M	70	Intermediate	Normal	Normal	70

BM, bone marrow; CK, complex karyotype.

**Figure 2:**
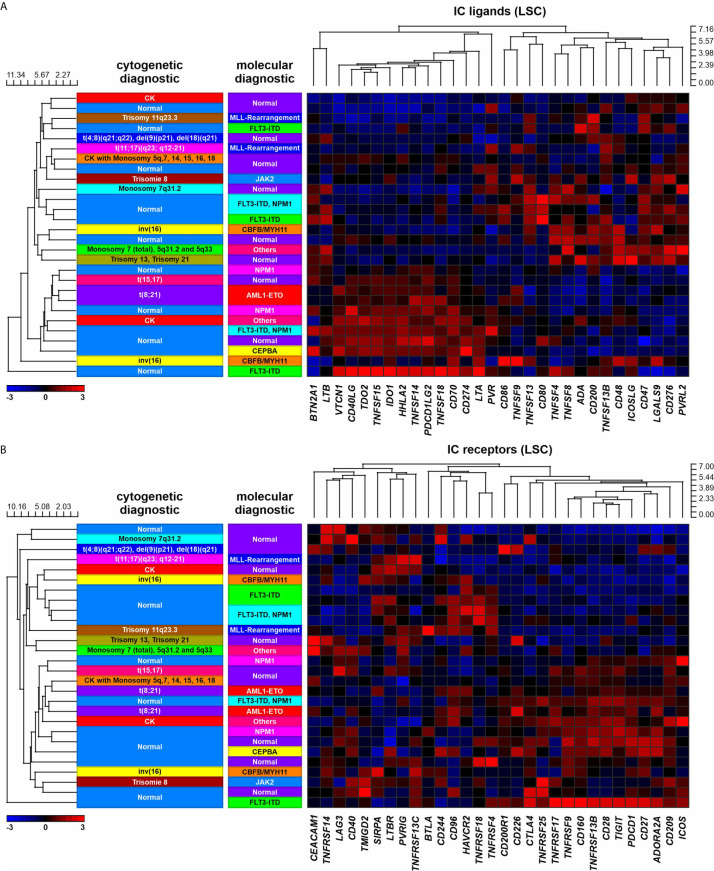
IC expression in LSCs in AML patients with different molecular or cytogenetic aberrations. **(A)** Heatmap indicating the gene expression profile of 30 IC ligands. **(B)** Heatmap indicating the gene expression profile of 30 IC receptors.

First, we analyzed the expression of 30 defined IC ligand/receptor genes in LSCs and LPCs as well as in control hematopoietic stem cells (HSCs) and control hematopoietic progenitor cells (HPCs). We grouped the ligands and receptors as activating or inhibitory ICs based on their main reported function, although many ICs have activating or inhibitory functions depending on the cell type and receptor signaling. Principal component analysis (PCA) revealed a distinct gene expression pattern between most LSCs/LPCs compared to the control HSCs/HPCs (**Figure 1B**). LSCs/LPCs and HSCs/LPCs expressed the majority of the analyzed IC ligand/receptor genes (**Figures 1C–F**). Ten IC ligands and 11 receptors were significantly upregulated in LSCs/LPCs compared to HSCs/HPCs. The gene expression of IC ligands and receptors was independent of the AML risk groups (**Figure 2** and **Supplementary Figure 1**). The number of upregulated IC genes in LPCs was higher than in LCSs (**Figures 1C, F** and **Supplementary Figure 2, 3**). Gene set enrichment analysis (GSEA) of the 30 analyzed IC ligand/receptor pairs confirmed the upregulation of IC ligands and receptors (**Figures 1D, G**). Interestingly, many upregulated genes in LSCs/LPCs encode for the IC ligands/receptors with an inhibitory function (e.g., *CD200*, *CD276*, *IDO1*, *PDCD1LG2*, *TDO2*, *VTCN1*, *CD96*, *CD160*, *CTLA4* and *HAVCR2*). A high gene expression of IC ligands/receptors with an activation function or a low expression of ICs with inhibitory function in LSCs correlated with improved overall survival (**Figure 1E**). A similar trend was observed for IC genes expressed on LPCs without reaching significance (**Supplementary Figure 3B**).

### IC Ligands Are Upregulated While Receptors Are Downregulated in BM-Infiltrating T Cells

**Figure 3:**
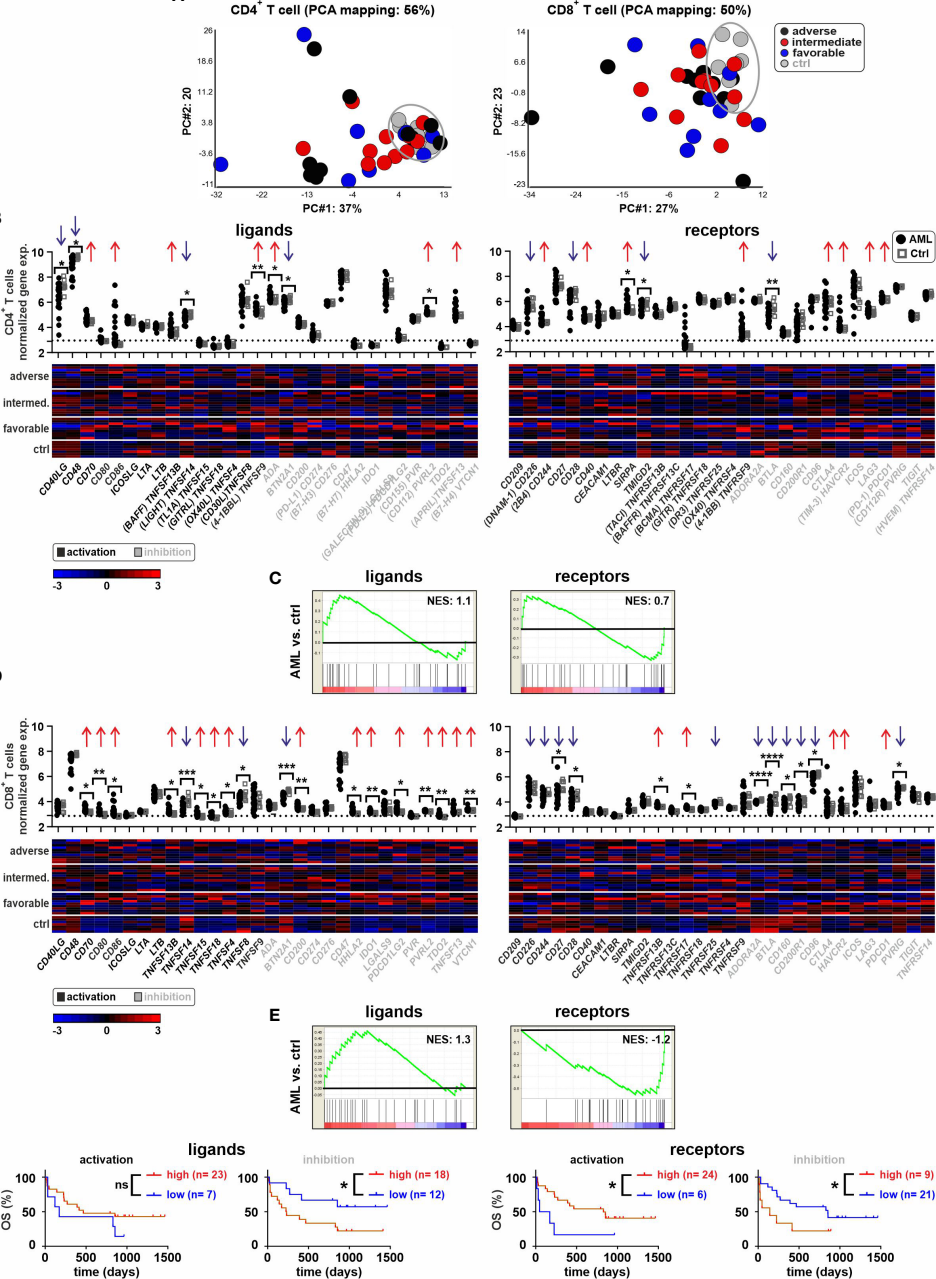
Expression of ICs in BM-derived T cells. **(A)** PCA of CD4^+^ or CD8^+^ T cells from AML patients and controls. **(B)** Gene expression patterns of ICs in CD4^+^ T cells from leukemia patients compared to controls (30 ligands and 30 receptors). Dot plots indicate the gene expression profiles (red arrows showing upregulated ICs and blue arrows indicating downregulated ICs; Fold difference ≥ 1.3). Heatmaps illustrated the gene expression patterns in respective AML risk groups. **(C)** GSEA representing the normalized enrichment score (NES) of IC gene sets on CD4^+^ T cells (AML vs. ctrl). **(D)** Gene expression patterns of ICs in CD8^+^ T cells from leukemia patients compared to controls. Dot plots show the gene expression profiles and heatmaps illustrate the expression patterns in respective AML risk groups. **(E)** GSEA representing the NES of IC gene sets on CD8^+^ T cells (AML vs. ctrl). **(F)** Kaplan–Meier plots of overall survival (OS) for AML patients in the study cohort according to the gene expression signature of activating or inhibitory ICs in CD8^+^ T cells. Statistics: student’s t-test and log-rank test. *P < 0.05, **P < 0.01, ***P < 0.001, ****P < 0.0001, ns, not significant.

Next, we analyzed the IC gene expression profile of CD4^+^ and CD8^+^ T cells in the BM (**Figure 3**). The PCA analysis revealed a distinct gene expression pattern between BM-infiltrating CD4^+^ and CD8^+^ T cells in leukemia as compared to the controls (**Figure 3A**). Six out of 30 IC ligand genes and three of 30 IC receptor genes were differentially expressed in CD4^+^ T cells of leukemia patients (**Figures 3B, C** and **Supplementary Figure 4**). In CD8^+^ T cells from leukemia patients, 17 of 30 ligands and 10 of 30 receptors were differentially expressed. IC ligand genes were preferentially upregulated, whereas, the receptor genes were mainly downregulated in CD8^+^ T cells from AML patients (**Figures 3D, E**, **Supplementary Figure 5**). Similarly, a high gene expression of ICs with either activating function or a low expression of IC genes with inhibitory function in CD8^+^ T cells of AML patients correlated with improved overall survival (**Figure 3F**). In contrast, the expression of IC genes in CD4^+^ T cells was not a prognostic marker (**Supplementary Figure 4B**).

### IC Receptors in AML BM-Infiltrating T Cells Are Silenced Due to Pathological Histone Deacetylation

**Figure 4:**
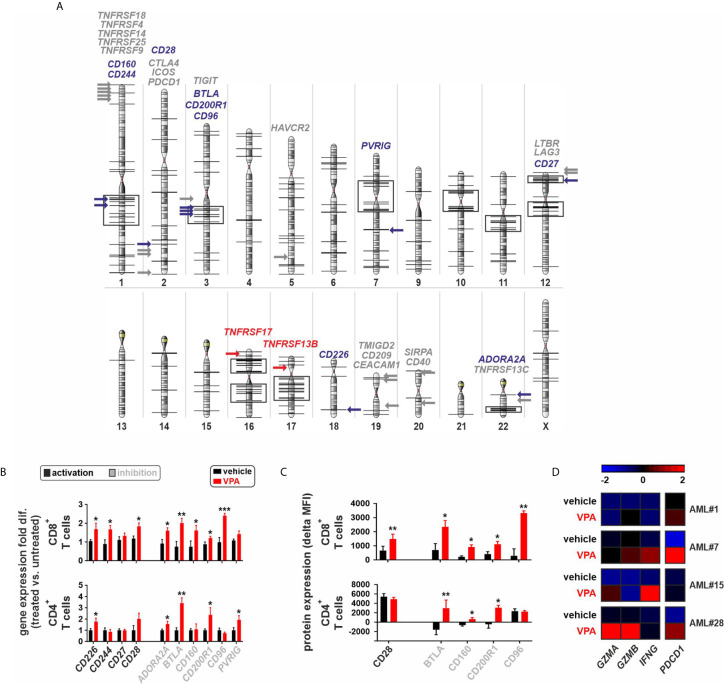
Epigenetic silencing of immune-checkpoint receptors in BM-infiltrating T cells. **(A)** Chromosomal position-based gene-mapping (Karyogram) analysis indicating the localization of 30 IC receptors. Black vertical lines on the chromosomal surfaces are indicating the previously identified downregulated genes in CD8^+^ T cells that were enriched in particular chromosomal regions mainly due to histone deacetylation (12). Black boxes indicate the chromosomal regions containing at least 5 downregulated genes located in a genome distance with less than 5 Mbp from each other, as potential hotspots for the histone remodeling. The 30 newly analyzed IC receptors of AML CD8^+^ T cells are plotted and highlighted with colored arrows in the karyogram to assess their chromosomal localizations (downregulated ICs: blue arrows; upregulated ICs: red arrows; ICs with no change: gray arrows). **(B)** Gene expression profile of 10 downregulated IC receptors in CD8^+^ or CD4^+^T cells of AML patients upon treatment with VPA. The fold differences were calculated as the ratio of VPA vs. vehicle conditions (n=5 AML patients per each group of CD8^+^ T cells and n=4 AML patients per each group of CD4^+^ T cells). **(C)** Surface protein expression of IC receptors on CD8^+^ or CD4^+^ T cells of AML patients. Delta MFI: MFI staining - MFI isotype (n=4 AML patients per group). **(D)** Heatmap illustrates the expression patterns of three key genes regulating the function of CD8^+^ T cells as well as *PDCD1* gene expression, upon treatment with VPA (n=4 AML patients). Statistics: student’s t-test. *P < 0.05, **P < 0.01, ***P < 0.001.

Since IC receptor genes were predominantly downregulated in CD8^+^ T cells of AML patients, we postulated that this phenomenon may potentially be due to chromatin remodeling. Previously, we documented a general downregulated gene signature in BM derived CD8^+^ T cells in AML and an upregulation of genes regulating chromatin organization or negatively regulating gene expression and transcription (12). In addition, chromosomal position-based gene-mapping (Karyogram) analysis predicted that downregulated genes were mainly enriched in defined genome regions as indicative of aberrant histone deacetylation (12). In CD8^+^ T cells, Karyogram analysis of IC receptors revealed that the majority of downregulated IC receptor genes (*BTLA*, *CD27*, *CD96*, *CD160*, *CD200R1*, and *CD244*) were localized in the predicted hotspots for histone remodeling. In contrast, upregulated IC receptor genes or those that were similarly expressed in AML patients and controls, were distributed in other chromosomal regions (**Figure 4A**).

To assess the impact of histone deacetylation on the gene expression of downregulated IC receptors at functional levels, FACS-purified CD8^+^ or CD4^+^ T cells from different AML patients with downregulated gene expression of IC receptors were treated with the histone deacetylase inhibitor (HDACi), Valproic acid (VPA). Treatment with VPA significantly increased the expression of IC receptors at both mRNA and protein levels (**Figures 4B, C**, **Supplementary Figure 6**). In addition, VPA treatment increased the gene expression of granzyme A (*GZMA*), granzyme B (*GZMB*), interferon-gamma (*IFNG*), and *PDCD1* in CD8^+^ T cells (**Figure 4D**).

**Figure 5:**
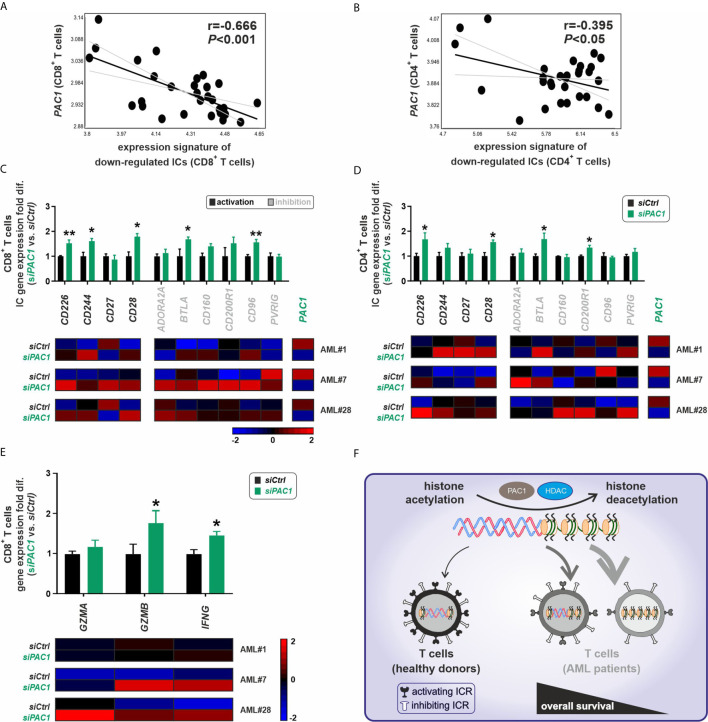
Epigenetic silencing of immune-checkpoint receptors in T cells is modulated *via* PAC1. **(A, B)** Correlation analysis of the gene expression signature for downregulated ICs vs. expression pattern of *PAC1* gene, in CD8^+^ or CD4^+^ T cells respectively (n=30 AML patients). **(C, D)** Gene expression profile of 10 downregulated IC receptors in CD8^+^ or CD4^+^T cells of AML patients upon *PAC1* gene silencing. The fold differences were calculated as the ratio of *siPAC1* vs. *siCtrl* (n=3 AML patients per each group of CD8^+^ or CD4^+^ T cells). **(E)** Expression profile of key effector molecules of CD8^+^ T cells upon *PAC1* gene knockdown (n=3 AML patients). **(F)** BM-infiltrating T cells in AML are dysfunctional due to a downregulation of activating IC receptors mainly *via* a pathologic epigenetic silencing through histone deacetylation; ICR, immune checkpoint receptor; HDAC, histone deacetylase. Statistics: student’s t-test. *P < 0.05, **P < 0.01.

The phosphatase of activated cells 1 (PAC1; also known as dual specificity phosphatase 2, DUSP2) was recently identified as a T cell suppressor and a crucial epigenetic immune regulator that acts *via* the histone-deacetylase complex leading to chromatin remodeling of effector T cells (13). In our AML patient cohort, *PAC1* gene expression negatively correlated with the gene expression of downregulated IC receptors, particularly in CD8^+^ T cells and partially in CD4^+^ T cells (**Figures 5A, B**). To assess the functional role of *PAC1* on the expression of downregulated IC receptors, we silenced the *PAC1* gene in FACS-purified CD8^+^ or CD4^+^ T cells from AML patients with downregulated gene expression signature of IC receptors. Knockdown of *PAC1* gene resulted in a significant upregulation of different IC genes (**Figures 5C, D**). Importantly, in CD8^+^ T cells, knockdown of *PAC1* led to a significant upregulation of *GZMB* and *IFNG* genes suggesting an improved functionality of CD8^+^ T cells (**Figure 5E**).

These findings indicate that silenced expression of IC receptors in AML BM-infiltrating T cells is mainly due to pathological histone deacetylation and can be reversed by treatment with deacetylation inhibitor agents or by blocking the T cell-specific epigenetic regulator PAC1 (**Figure 5F**).

## Discussion

Dysfunctional and/or immunosuppressive T cells in AML have been previously described, specifically in peripheral blood (14–18). AML LSCs express different co-stimulatory and co-inhibitory ligands, and the efficacy of ICIs is identifiably lower in leukemia than in solid tumors (11). Although the role of the PD-1/PD-L1 pathway in immune evasion has been reported in several murine leukemia models (19–21), clinical trials with PD-1 blocking antibodies as monotherapy have revealed a low response rate in patients. Several studies with different PD-1 blocking antibodies are ongoing, either at a time point when most of the leukemia bulk has been eliminated prior to chemotherapy, in combination with chemotherapy, or in combination with hypomethylating agents that upregulate the expression of PD-L1 on leukemia cells (11). In addition, tri-specific antibodies that combine T cell redirection to CD33 expressing myeloid cells together with an anti-PD-L1 part demonstrate enhanced cytotoxicity against primary AML cells *in vitro* (22). Non responding patients to PD-1 blockade, often co-express other immune inhibitory receptors, such as TIM-3 and LAG-3 on effector T cells (11). Furthermore, AML blasts expressing T cell inhibitory ligands B7-H3, galectin-9 (Gal-9), and CD200. The expression of most of these ligands is not limited to a given FAB subtype. However, TP53-mutated AML more frequently expresses PD-L1 and 4-1BBL (23).

Leukemia cells do not only express ligands for immune-checkpoints that regulate the leukemia-specific immune response but also express immune-checkpoint receptors (24). This implies that niche cells (including immune cells) that express ligands for these co-stimulatory or co-inhibitory receptors, may regulate LSC function (25). The exact mechanism of how the expression of these co-stimulatory molecules induces AML disease progression and poor prognosis remains unclear. To analyze the interactions of IC ligands and receptors in AML, we performed a detailed gene expression analysis in well-defined samples of FACS-purified leukemia stem/progenitor cells and paired BM-infiltrating CD4^+^ and CD8^+^ T cells from AML patients and controls.

Our data indicate that ICs are dysregulated on AML LSCs/LPCs as well as in BM-infiltrating T cells. This promotes multiple interactions between the individual cell populations, leukemia, and immune cells. IC receptor and ligand genes were mainly upregulated in LSCs and LPCs. Some of these ICs, such as CTLA-4, LAG-3, CD27, LTbr, TIM-3, and its ligand Gal-9 or co-stimulatory molecules, like CD80, CD86, CD40, have been shown to expand LSCs and contribute to disease progression (26–28). In the present study, we did not analyze the role of individual ICs but rather grouped the ligands and receptors as activating or inhibitory ICs based on their main reported function, mainly in T cells (5). However, the function of ICs activation vs. inhibition depends on the cell type and the context of the signaling (5, 29, 30). Interestingly, the gene expression of activating IC ligands and receptors correlated with improved survival of AML patients, whereas, the gene expression of inhibitory IC ligands or receptors was a negative prognostic factor. The comparison to control HSC/HSPCs revealed that mainly inhibitory ligand genes were upregulated. This suggests that the sum of the inhibitory signals provided to T cells diminishes the anti-leukemic immune response. This inhibitory microenvironment in the bone marrow may be further increased by the expression of most of the inhibitory IC ligands, including, PD-L1 on BM-infiltrating CD8^+^ T cells. However, we observed a strong downregulation of most IC receptor genes on BM-infiltrating CD8^+^ T cells, possibly reducing the ligand/receptor interaction. PD1 seems to be one exception and Daver et al. recently reported that the frequency of PD1^+^/CD8^+^ T cells was significantly higher in bone marrow aspirates (BMAs) of patients with newly diagnosed AML than in healthy BMAs as controls. A similar but not significant difference was observed for the frequency of OX40^+^/CD8^+^ T cells (23). Comparably, some IC receptors, including PD-1, were upregulated in CD4^+^ or CD8^+^ T cells of AML patients versus healthy controls in the present study.

Different studies have demonstrated that aberrant epigenetic alterations, such as histone remodeling and DNA methylation play a crucial role in the dysregulation of ICs during carcinogenesis (14, 15, 31). In addition, DNA hypomethylating agents (HMAs) such as azacitidine and decitabine upregulate the expression of PD-L1 in solid tumors (16) and AML (17) and thereby dampen the anti-tumor immune response. A clinical study testing the combination of azacytidine in combination with the PD-1 blocking antibody nivolumab in the treatment of AML resulted in an overall response rate of 33% (58% for the HMA-naïve patients (18)). We recently documented that pathological epigenetic alteration *via* histone deacetylation silences gene expression in BM-infiltrating CD8^+^ T cells. This silenced gene expression was a positive prognostic factor and CD8^+^ T cells supported the maintenance and expansion of (12). This finding was somewhat counterintuitive, as activated CD8^+^ T cells are necessary to eliminate LSCs. The preferential downregulation of IC receptors in AML BM-infiltrating CD8^+^ T cells suggests that they are regulated by epigenetic mechanisms. This hypothesis was strengthened by the preferential localization of the downregulated IC receptors in predefined hotspots for histone remodeling.

Histone deacetylase (HDAC) enzymes modulate maturation, migration, and TCR signaling in T cells by mediating the expression of essential transcription factors such as Tcf1 and Lef1 (32, 33). Treatment with HDACi enhances the anti-tumor activity of cytotoxic T cells in different tumor types (34). In addition, HDACi directly acts on tumor cells by upregulating MHC class I molecules and indirectly by eliminating myeloid-derived suppressor cells (35). Notably, HDACis can increase the expression of immune-related molecules in different cancer types (36, 37). In our study, treatment with HDACi increased the gene expression of defined IC receptors mainly in AML BM-infiltrating CD8^+^ T cells and partially in CD4^+^ T cells.

Several studies have demonstrated that treatment with HDACi dampens the anti-tumor immune response by upregulation of PD-1 on T cells (38, 39). Thus, PD-1/PD-L1 blocking antibodies and HDACis have synergistic effects in the treatment of different cancers (38, 39). Additionally, VPA treatment blocked the function of myeloid-derived suppressor cells and made tumor cells susceptible to anti-PD-L1 immunotherapy (40). Similarly, treatment with HDACi in our study resulted in an upregulation of *PDCD1* in CD8^+^ T cells, suggesting an increased vulnerability to treatment with ICIs

The phosphatase PAC1 was recently identified as an important mediator of T cell dysfunction in tumor infiltrating lymphocytes (TILs) that act *via* histone deacetylation to reshape chromatin accessibility during activation of effector T cells (13). We observed a negative correlation of *PAC1* expression with the expression of the downregulated ICs in AML BM-infiltrating CD8^+^ T cells. Silencing of *PAC1* resulted in the upregulation of different ICs and enhanced the expression of CD8^+^ T cell effector molecules.

In summary, our results indicate that CD8^+^ T cells in AML are dysfunctional due to a downregulation of activating IC receptors rather than due to signaling by immune inhibitory IC receptors. These findings may explain the limited efficacy of antibodies that block immune-inhibitory ICs. Therefore, treatment with HDACi or targeting of T cell-specific epigenetic modulators may stimulate anti-tumor immunity by increasing CD8^+^ T cell effector function in leukemia patients and restore the susceptibility to treatment with IC blocking agents.

## Data Availability Statement

All transcriptomic data compiled for this study have been deposited in NCBI GEO under the accession codes: GSE117090.

## Ethics Statement

The studies involving human participants were reviewed and approved by the local ethical committee (Kantonale Ethikkommission Bern, KEK122/14). The patients/participants provided their written informed consent to participate in this study.

## Author Contributions

RR designed and performed experiments, analyzed and interpreted data, and wrote the manuscript. MS performed the experiments. CR designed the experiments and interpreted data. AO designed experiments, interpreted data, wrote the manuscript, and supervised the project. All authors contributed to the article and approved the submitted version.

## Funding

This work was supported by grants from the Werner und Hedy Berger-Janser Stiftung, Swiss National Science Foundation and the Stiftung für klinisch-experimentelle Tumorforschung (Bern).

## Conflict of Interest

The authors declare that the research was conducted in the absence of any commercial or financial relationships that could be construed as a potential conflict of interest.
