# Supplementary material for: Epigenetic Silencing of Immune-Checkpoint Receptors in Bone Marrow- Infiltrating T Cells in Acute Myeloid Leukemia
Source: Front Oncol. 2021 May 4;11:663406. doi: 10.3389/fonc.2021.663406 (PMC8130556; doi:10.3389/fonc.2021.663406)
Supplement: Supplementary file 1 [file DataSheet_1.zip › Supplementary Material.pdf]

Epigenetic silencing of immune-checkpoint receptors in bone marrow-infiltrating T cells in acute myeloid leukemia

Ramin Radpour[1,2], Miriam Stucki[1,2], Carsten Riether[1,2], Adrian F. Ochsenbein[1,2]

[1] Tumor Immunology, Department for BioMedical Research (DBMR), University of Bern, Bern, Switzerland.

[2] Department of Medical Oncology, Inselspital, Bern University Hospital, University of Bern, Bern, Switzerland.

Correspondence:

Ramin Radpour, Department of Medical Oncology, Inselspital, Bern University Hospital,3010 Bern, Switzerland. E-mail: ramin.radpour@dbmr.unibe.ch, Telephone: +41-31-632-0956, Fax: +41-31-632-4119.

Adrian F. Ochsenbein, Department of Medical Oncology, Inselspital, Bern University Hospital,3010 Bern, Switzerland. E-mail: adrian.ochsenbein@insel.ch, Telephone: +41-31-632-4114, Fax: +41-31-632-4119.

Supplementary Figures

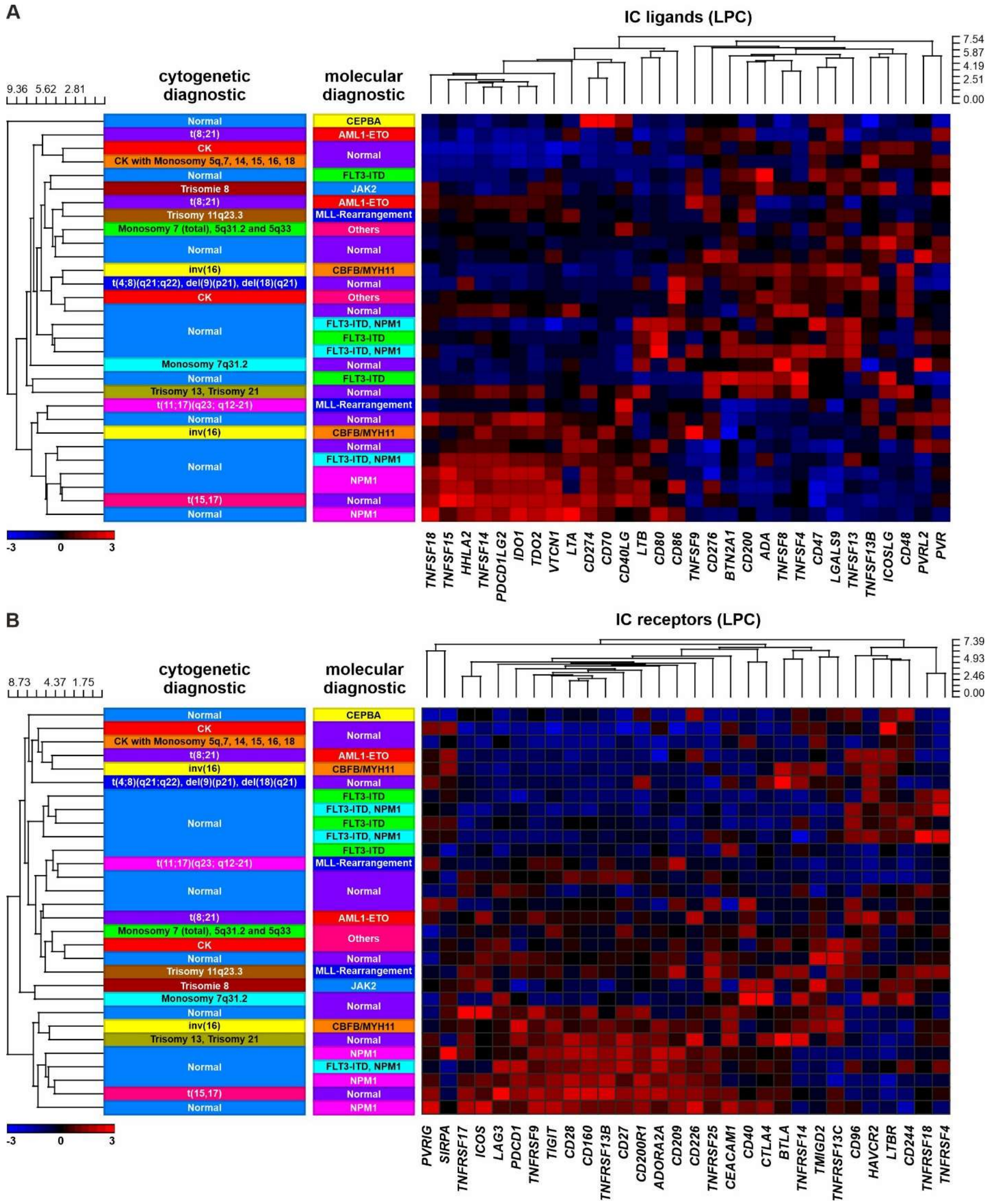

Supplementary Figure 1 Correlation of the IC expression in LPCs with known molecular or cytogenetic aberrations. **(A)** Heatmap indicating the gene expression profile of 30 IC ligands. **(B)** Heatmap indicating the gene expression profile of 30 IC receptors.

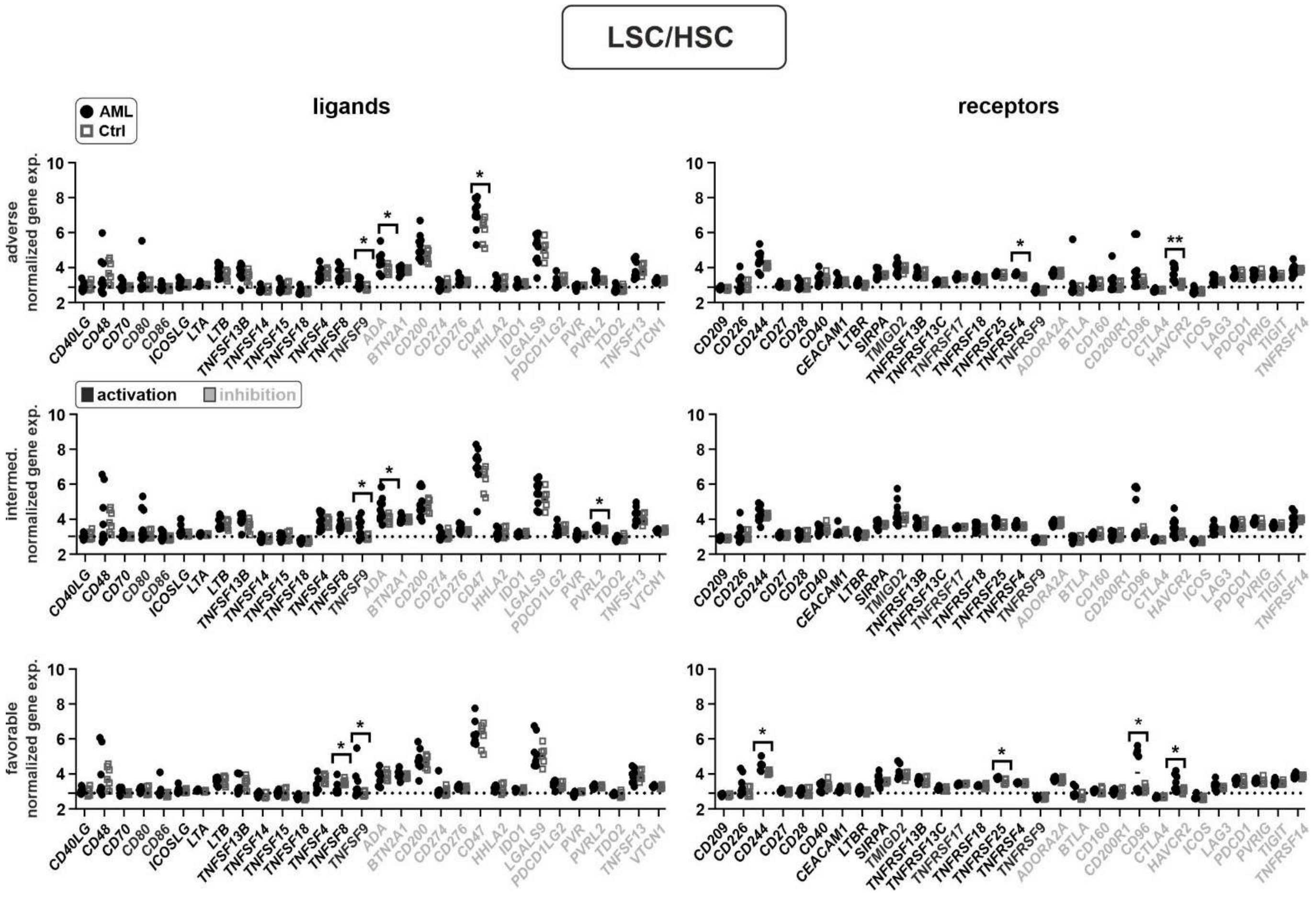

Supplementary Figure 2 Gene expression signature of IC ligands/receptors in LSCs across AML risk groups. Statistics: Student's t-test. *P<0.05, **P<0.01, ***P<0.001.

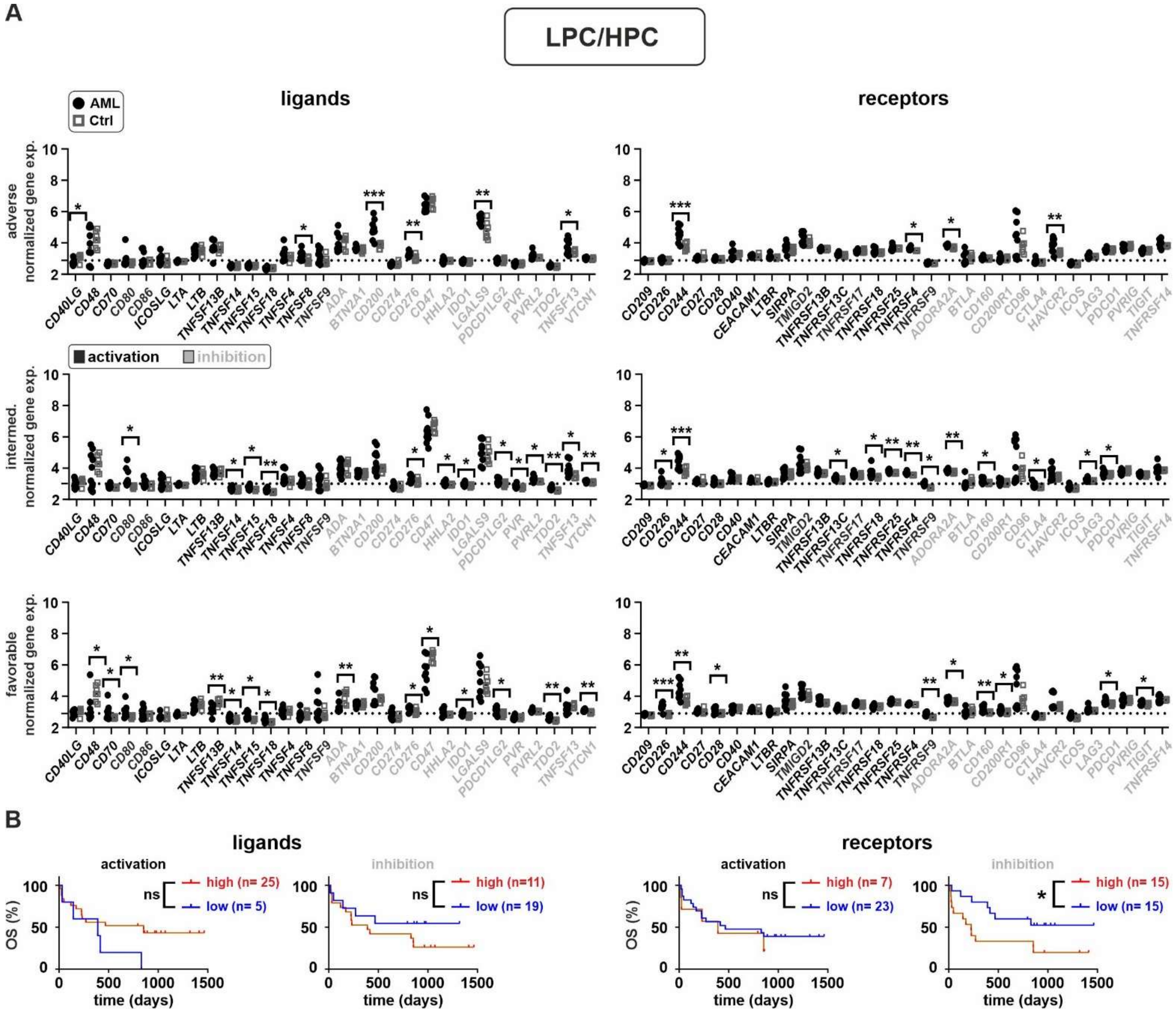

Supplementary Figure 3 (A) Gene expression signature of IC ligands/receptors in LPCs across AML risk groups. **(B)** Kaplan–Meier plots of overall survival (OS) for AML patients in the study cohort, according to gene expression profile of IC ligands/receptors for LPCs. Statistics: Student's t-test (A) and log-rank test (B). **P*<0.05, ***P*<0.01, ****P*<0.001.

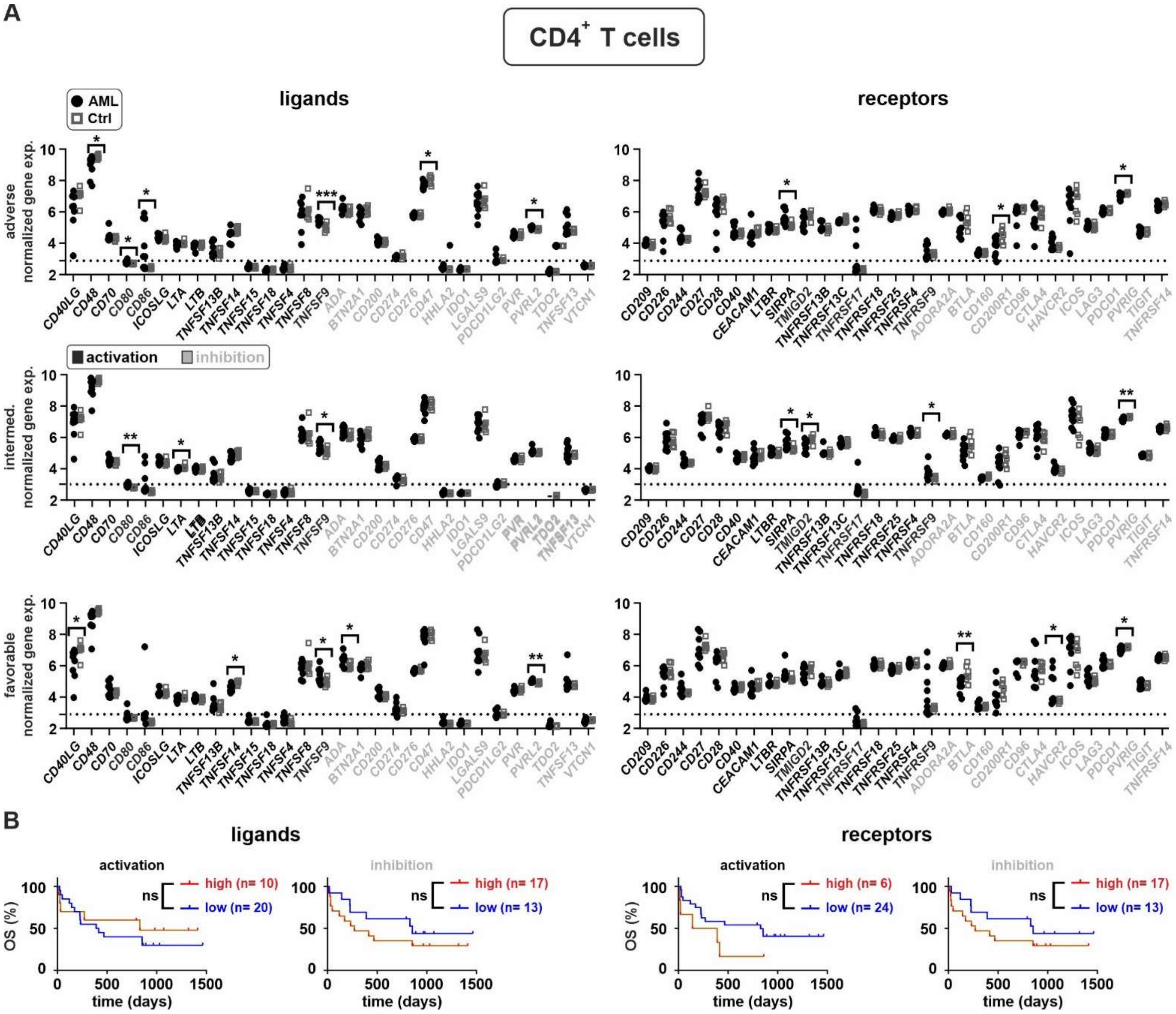

Supplementary Figure 4 (A) Gene expression signature of IC ligands/receptors in leukemia derived CD4+ T cells across AML risk groups. **(B)** Kaplan–Meier plots of overall survival (OS) for AML patients in the study cohort, according to gene expression profile of IC ligands/receptors for AML derived CD4+ T cells. Statistics: Student's t-test (A) and log-rank test (B). **P*<0.05, ***P*<0.01, ****P*<0.001.

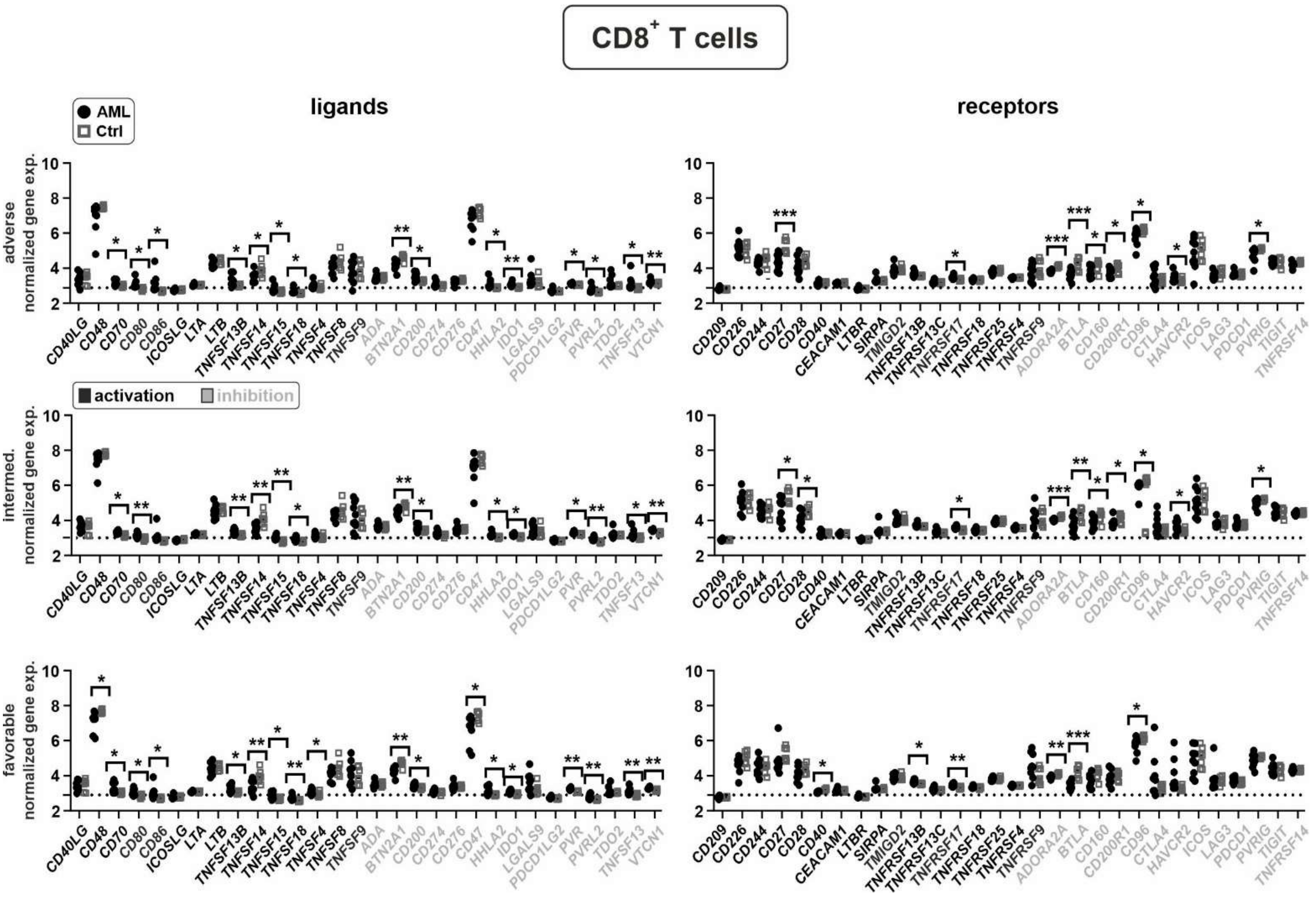

Supplementary Figure 5 Gene expression signature of immune-checkpoint ligands/receptors in leukemia derived CD8+ T cells across AML risk groups. Statistics: Student's t-test. *P<0.05, **P<0.01, ***P<0.001.

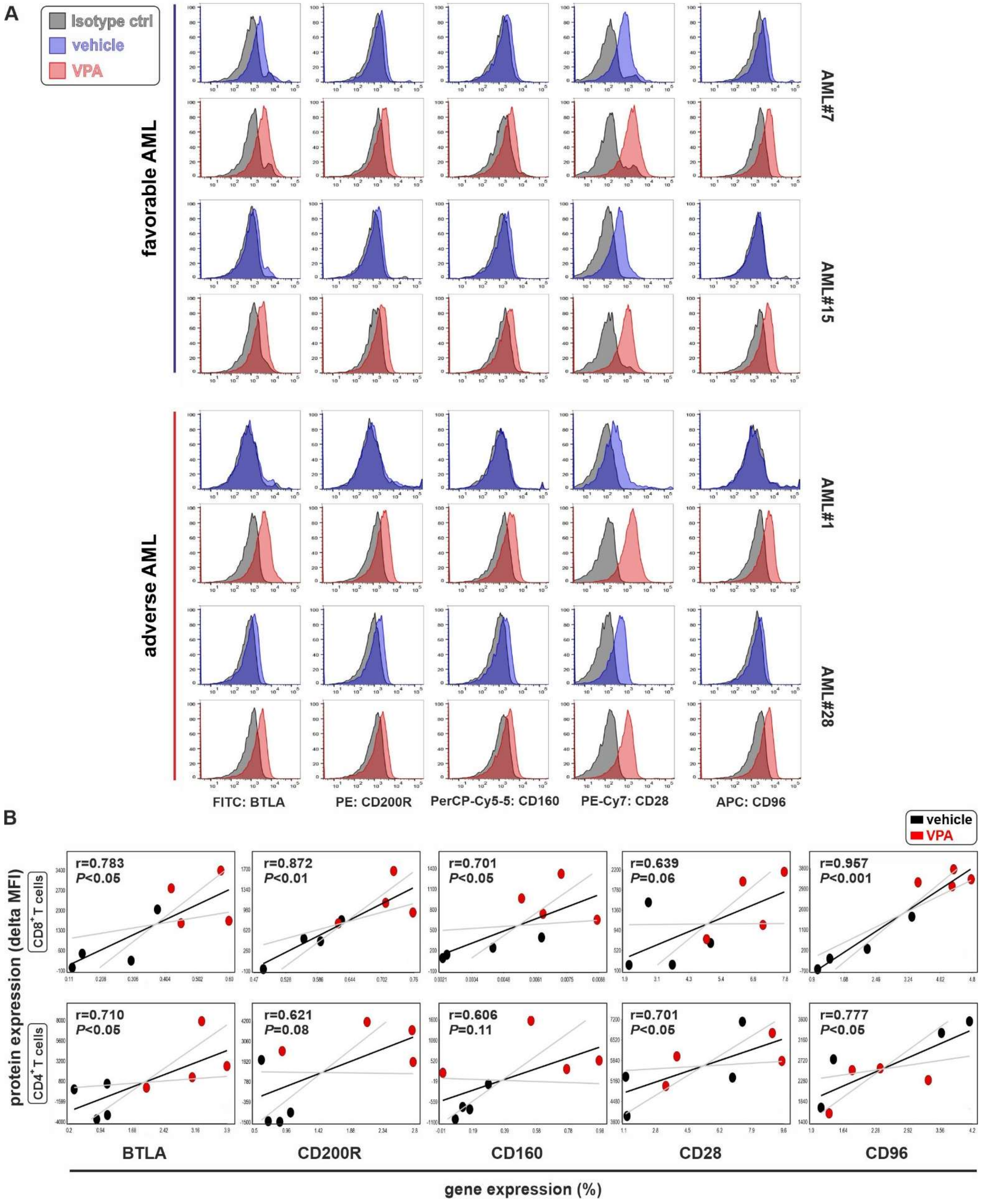

Supplementary Figure 6 (A) Protein expression profile of five selected downregulated IC receptors in CD8+ T cells of four AML patients. Analysis was performed on FACS-purified CD8+ T cells upon 48h treatment with VPA or vehicle. Correlation analysis of gene expression vs. protein expression of five different IC receptors (BTLA, CD200R, CD160, CD28 & CD96), upon treatment with VPA or vehicle (n=4 AML patients).

Supplementary Table 1 Primer sequences.

Gene name	Forward primer (5'→3')	Pos.	Tm	Reverse primer (5'→3')	Pos.	Tm	Amp. (bp)
ACTB*	GCACCACACCTTCTACAATGAG	449	61.7	GGTCTCAAACATGATCTGGGTC	569	61.2	120
ADORA2A	GCTCCATCTTCAGTCTCCT	690	58.5	CAAACGACAGCACCCAG	821	58.6	131
BTLA	TGAAGACATTGCCTGCCA	115	59.6	GTTAGCACAGTATTTCACAGGG	308	59.4	193
BTN2A1	TTCCAGAATCCTTTATGCCCAG	924	60.6	AGCAATTTCTCTTGTTTCCCGT	1096	61.2	172
CD27	CCGAGTGTGATCCTCTTCC	581	59.6	CCTCCAGCATCTCACTGAC	688	59.9	107
CD28	TTTCTGGGTGAGGAGTAAGAG	585	58.9	ATAGGCGTCCGTGTCAG	733	58.7	148
CD96	TACAACCTTCTCATTCAGACAC	524	58.2	GTTCCATTATCCTCCTTTATACCC	735	58.7	211
CD160	ATGTTCACCATAAGCCAAGTC	369	58.7	GTGTAGTTCCCTGTCTCTGTG	499	60.2	130
CD200R1	ACCTCCAAGTGTTAGTTACACCT	751	61.4	GTCACTGTGCCATTGCTCC	910	61.8	159
CD226	TCACATCTCAAGAACCAGCC	230	60.1	ACCTGTGTTAAGATGCCCA	411	59.1	181
CD244	ACCCTTGCCTGCTTCTG	812	59.6	CTCCTGCTCGTGATTTCTCC	940	60.6	128
GAPDH*	TCATTTCCTGGTATGACAACGA	1594	59.9	CTTCCTCTTGTGCTCTTGCTG	1714	61.7	120
GZMA	TCAGGTTGATTGATGTGGGAC	4	60.5	CAGACATCTTCAGGAATTAGCAGG	111	61.5	107
GZMB	CTGACTTACGCCATTATTACGAC	553	59.8	CCATAGGAGACAATGCCCTG	688	60.4	135
IFNG	GGGTTCTCTTGGCTGTTACTG	179	61.1	TTTCTGTCACTCTCCTCTTTCCA	328	61.5	149
PAC1 (DUSP2)	CCATAGGCTTCATTGACTGGG	801	60.9	GCTGCTTAACGAAGTCAAAGG	953	60.3	152
PDCD1	GCACGAGGGACAATAGGAG	641	60.0	CGCTAGGAAAGACAATGGTGG	821	61.5	180
PVRIG	GCTGACAACATGAAGACTTCCT	338	61.0	CCTCCATCCGAACTTGAACC	516	60.8	178
TNFSF8	TTTCTATTTGACCACAGCCACTC	227	61.3	GTCTTCTGAGCAATTTCCTCCT	368	60.5	141
TNFSF14	GATACAAGAGCGAAGGTCTCAC	630	60.9	TGAGTCTCCCATAACAGCGG	731	61.8	101
VTCN1	AGTCACCAAGGAAGGCAG	3	59.3	AGTGTCTCCCTGAAATACCA	177	58.1	174

* Reference gene
